# Variation in Systemic Antibiotic Treatment for Diabetic Foot Osteomyelitis in England and Wales: A Multi-Centre Case Review †

**DOI:** 10.3390/jcm13113083

**Published:** 2024-05-24

**Authors:** Akram Uddin, David A. Russell, Fran Game, Derek Santos, Heidi J. Siddle

**Affiliations:** 1 Northamptonshire Healthcare NHS Foundation Trust, Northamptonshire NN11 4DY, UK; 2 Essex Partnership University NHS Foundation Trust, Essex SS4 1RB, UK; 3 Leeds Institute of Clinical Trials Research, University of Leeds, Leeds LS2 9JT, UK; 4 Leeds Vascular Institute, Leeds Teaching Hospitals NHS Trust, Leeds LS9 7TF, UK; 5 University Hospitals of Derby & Burton NHS Foundation Trust, Derby DE22 3NE, UK; 6 School of Health Sciences, Queen Margaret University, Edinburgh EH21 6UU, UK; 7 Leeds Institute of Rheumatic and Musculoskeletal Medicine, University of Leeds, Leeds LS7 4SA, UK

**Keywords:** bone infection, diabetic foot osteomyelitis, diabetic foot infection, systemic antibiotics, oral antibiotics, intravenous antibiotics

## Abstract

**Background:** Diabetic foot osteomyelitis (DFO) is a major complication and can lead to significant morbidity and mortality. Systemic antibiotic therapy is often initiated first line to achieve quiescence of infection. To perform a multi-centre case review of systemic antibiotic intervention to treat adults with DFO in England and Wales and compare with national guidelines ‘*Diabetic foot problems: prevention and management*’. **Methods:** Eight centres from England and Wales retrospectively collated data from a minimum of five adults (aged ≥ 18 years) from electronic case records. All patients were treated with systemic antibiotics following a new diagnosis of DFO (1 June 2021–31 December 2021). **Results:** 40 patients (35 males and 5 females) were included; the mean age was 62.3 years (standard deviation (SD) 13.0). Patients commenced systemic oral 14 (35%) or intravenous 26 (65%) antibiotic therapy following a new diagnosis of DFO. Twenty-seven (67.5%) patients were medically or surgically managed in the 12-week period with clinical quiescence of infection. Twenty-one patients (52.5%) had no recurrence of DFO infection within 12 weeks; seventeen (42.5%) of these patients had clinical quiescence of infection with systemic antibiotics alone without surgical intervention and nine (22.5%) of these cases had no recurrence of DFO. There were no cases of major amputation or death. All centres showed significant in-centre variability in systemic antibiotic management; variability was reported in the clinical and quantity indicators specifically to antibiotic selection, single versus dual therapy, mode of delivery and duration of treatment. **Conclusions:** This case review identifies there is existing variation when treating adults with systemic antibiotics for DFO. Further national guidance is required to standardise service delivery and care to improve patient outcomes.

## 1. Introduction

Diabetes mellitus is on the rise globally and a major concern in healthcare worldwide [1]. In the United Kingdom (UK), its prevalence is increasing, with 5 million people estimated to have diabetes by 2025 [2]. During 2017–2020 a total of 171,759 diabetic foot disease-related hospital admissions were recorded resulting in 21,738 minor and 7957 major amputations [3].

As many as one in four patients with diabetes mellitus will develop a diabetic foot ulcer (DFU) during their lifetime [1,4]. It is estimated that up to 80% of DFUs will also develop a diabetic foot infection (DFI) and a third of these cases will potentially develop diabetic foot osteomyelitis (DFO) [5,6].

Osteomyelitis is an inflammatory process of bone and bone marrow caused by an infectious organism(s) resulting in destruction, necrosis, and apposition of new bone [7]. The International Working Group on the Diabetic Foot (IWGDF 2023) [8] guidelines identify systemic antibiotic therapy alone is as effective when treating DFO. DFO can be treated with systemic antibiotics, with and without surgical resection of the infected bone by a multidisciplinary diabetic foot team (MDFT) with substantive cost to the healthcare system [6,9].

DFU, as a typical consequence of the combination of peripheral neuropathy, trauma and/or peripheral artery disease (PAD), is frequently the entry point of pathogenic bacteria [2,10,11]. DFI may present superficially and if left untreated the pathogenic bacteria can spread through the subcutaneous layer and infect the deeper tissues and bone resulting in DFO [10,12]. DFI and DFO can lead to significant morbidity and mortality [4,13,14,15]. Early diagnosis and treatment may prevent DFO from becoming limb- and life-threatening; therefore, the control of infection in DFO is vital [1,12].

DFO should be diagnosed and classified defining the severity of infection using Infectious Diseases Society of America (IDSA)/IWGDF [8] criteria. The guidelines support six weeks of systemic antibiotics following identification of the causative microorganism when no resection has been performed [8,16]. The National Institute for Health and Care Excellence (NICE) [17] guidelines recommend systemic antibiotic therapy for a minimum period of six weeks when treating DFO. The NICE [17] guidance does not state a maximum time limit of systemic antibiotics when treating DFO and only recommends a course length is based on “*clinical assessment*”.

The optimal selection and duration of systemic antibiotic therapy when treating DFI/DFO is not defined [1,5]. There are no proven laboratory tests or imaging techniques to determine when systemic antibiotics should be discontinued [12]. Recurrence or reactivation is observed in up to 31% of cases when DFO is treated with systemic antibiotics alone [18]. The duration of systemic antibiotic therapy therefore varies with clinical and radiological responses to the intervention and quiescence of infection [19]. Studies have reported comparing 3 weeks with 6 weeks, and 6 weeks with 12 weeks of systemic antibiotic therapy for DFO with no significant differences when outcomes were compared [20,21]. A recent meta-analysis reported the medical management of DFO with systemic antibiotics alone ranged from 4 to 36 weeks [22].

Various antibiotics with different spectrums and routes of administration exist but the most effective regimen to treat DFO is not identified [5,23,24]. This multi-centre case review aims to identify current clinical practice of systemic antibiotics to treat adults with DFO by NHS MDFT in England and Wales.

## 2. Methods

**Figure 1:**
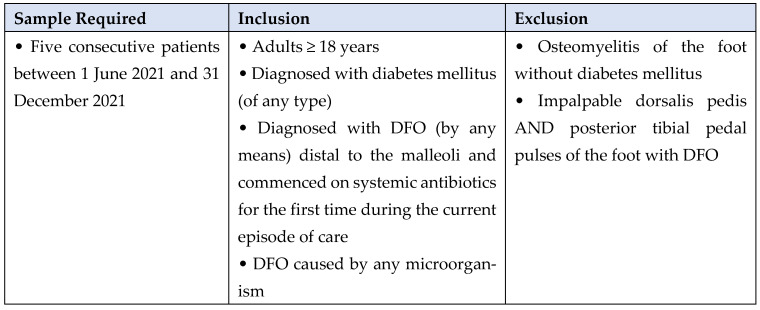
Case review criteria.

Clinical data were collected from eight MDFT centres using electronic and paper medical case records of five consecutive patients presenting between 1 June 2021 and 31 December 2021 who met the inclusion/exclusion criteria for the case review (Figure 1).

Each MDFT centre extracted data in electronic format following a standard operating procedure (SOP). The SOP ensured a consistent method of identifying adults with DFO and data extraction. 

The SOP was piloted by conducting the data extraction at MDFT centre 1. This was to verify the method of data collection was feasible and extracted the data required. Data were then extracted from MDFT centre 2 from a further five patients’ records by a second data collector to ensure the SOP was feasible for other centres to conduct comparable data collection. Each remaining participating centre was then instructed to follow the same process. Data were collected from the initial point of diagnosing DFO and initiating systemic antibiotic therapy (oral or intravenous) and for the following 12-week period. All MDFT centres collected their data from the same 6-month period (1 June 2021–31 December 2021).

**Figure 2:**
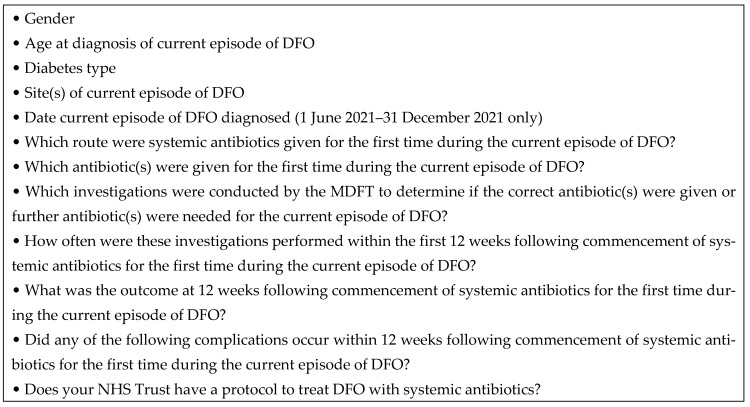
Data collected from all eight participating MDFT centres.

Each participating NHS centre registered the case review through their relevant audit approval process. The data were electronically collated and descriptive statistical methods were applied to report systemic antibiotic intervention to treat DFO and the outcomes over a 12-week period (Figure 2).

## 3. Case Review Standards

**Figure 3:**
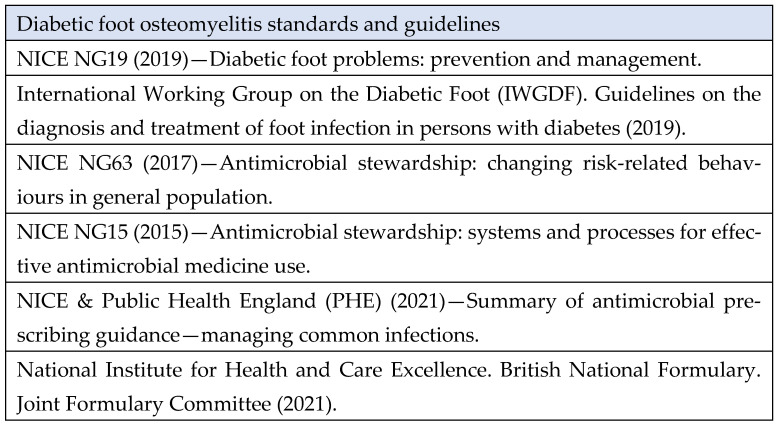
Case review standard guidelines [17,25,26,27,28,29].

Clinical indicators were selected after reviewing the relevant guidelines (Figure 3) and experience of the authors. These guidelines were in existence during the time of treatment (1 June 2021–31 December 2021). The guidelines determine the clinical standards for treating DFO in adults with systemic antibiotic therapy. The management of DFO in adults (aged ≥ 18) in England and Wales is based on NICE [17] guidelines where local policies, protocols and guidelines are developed to establish best practice. This case review compares the management of DFO with systemic antibiotics with the NICE [17] guidelines.

## 4. Results

Data were extracted from the medical care records of 40 (100%) patients diagnosed with DFO and treated with systemic antibiotic therapy from eight MDFT centres (Table 1). Demographic data, diabetes type and site of DFO for all 40 (100%) patients and for the patients at each MDFT centre are presented in Table 1.

Each MDFT centre prescribed oral and intravenous (IV) antibiotic regimes to treat DFO with variations in treatment modalities; only one centre (MDFT centre 2) treated all five (100%) cases with oral antibiotic therapy (Table 2). Ten (25%) patients were prescribed single antibiotic oral therapy and ten (25%) delivered single antibiotic IV therapy. Twenty (50%) patients were prescribed dual antibiotic therapy. Of those who had dual antibiotics, 16 (40%) had oral and 4 (10%) had IV antibiotics (Table 3).

The most frequent prescribed oral antibiotics (Table 3) were clindamycin and ciprofloxacin in nine (22.5%) patients, co-amoxiclav and amoxicillin in four (10%) patients, flucloxacillin and fusidic acid in three (7.5%) patients and flucloxacillin in three (7.5%) patients. The most prescribed IV antibiotics were co-amoxiclav accounting for three (7.5%) patients and teicoplanin for two (7.5%) patients (Table 3). Five out of eight MDFT centres reported having a protocol to treat DFO.

The radiological tests performed and frequency were also reviewed (Table 4 and Table 5).

The haematological and biochemical investigations performed, and frequency were also reviewed (Table 6 and Table 7). 

Clinical assessments and interventions from each MDFT centre and methods to obtain culture to support systemic antibiotic therapy prescribing were also reviewed over 12 weeks (Table 8). The frequency of these clinical assessments, interventions and tests were also reviewed (Table 9).

There were no reported deaths or major amputations during the 12-week period. Twelve (30%) patients underwent minor amputation and six (15%) patients had surgical debridement. Three (7.5%) of those treated surgically had an orthobiological agent impregnated with antibiotics. At 12 weeks, 12 patients (30%) continued with systemic antibiotics. The total number of patients who had complete quiescence of DFO was 27 (67.5%), and from this group, 5 (12.5%) remained on systemic antibiotic therapy at 12 weeks. Seventeen (42.5%) patients had complete quiescence of DFO with systemic antibiotics alone without surgical intervention, of which nine patients (22.5%) had no recurrence of DFO within the 12-week period (Table 10 and Table 11). 

Overall, 21 patients (52.5%) in total who were medically or surgically treated had no recurrent DFO in the 12-week period. Four centres (MDFT 1, 2, 3 and 4) reported 16 (40%) cases of recurrent DFO after systemic antibiotic therapy with 6 (15%) cases undergoing debridement or minor amputations (Table 10 and Table 11).

A total of 15 of 40 patients (37.5%) developed a complication during 12-week systemic antibiotic therapy when treating DFO (Table 12). 

## 5. Discussion

This multi-centre clinical case review focussed on identifying how adult patients (≥18 years) with DFO were treated with systemic antibiotic therapy and the investigations that were performed to measure clinical effectiveness. There was significant inter-centre variability in the clinical and quantity indicators: (1) route of systemic antibiotic administration; (2) antibiotic selection; (3) single or dual antibiotic therapy; (4) imaging investigation to determine effectiveness of systemic antibiotic therapy; (5) laboratory investigation to determine effectiveness of systemic antibiotic therapy; (6) clinical assessments. Variability in clinical practice is expected; however, the findings from this case review indicate that certain aspects of care can be standardised when treating DFO with systemic antibiotics. 

The NICE [17] guidelines clearly state that microbiological samples for culture and sensitivity should be obtained prior to, or as close as possible to commencing systemic antibiotic therapy. A soft tissue or bone sample is recommended and only if this cannot be obtained a deep wound swab should be taken. The greatest number of tests performed in this case review were wound swabs and accounted for 88 (17.5%) laboratory and clinical tests (Table 9). No MDFT centre performed fluid aspiration (0%) or thermographic scans (0%) (Table 8) and these tests are not identified diagnostic investigations when treating DFO according to NICE [17]. There is a requirement of minimum standards for diagnostic consistency in identifying a microorganism when DFO is suspected. Tissue samples were only performed in 14 (35%) (range 0–80%) cases and this practice was only undertaken in four MDFT centres (Table 8). Similar findings were observed for bone samples for MCS where four MDFT (MDFT centres 2, 3, 6 and 8) did not perform this investigation for any of their patients (Table 8). MDFT centre 8 reported one (2.5%) bone sample for histological examination to confirm osteomyelitis (Table 8). Bone culture for microscopy, culture and sensitivity (MCS) is considered as a sensitive diagnostic test for DFO [30] However, a bone culture was only obtained from eight (20%) patients in this case review (Table 8). The identified variations can be addressed by following specific guidance and standards on obtaining a bone sample or deep tissue sample when DFO is suspected [8,17]. This case review identified NICE [17] guidance was not followed by some centres that did not obtain a tissue sample or bone sample when treating DFO. Bone or deep tissue samples should be obtained, and training may be required to ensure MDFT clinicians are skilled to perform this. The practice of repeated culture tests needs to be based on reason and performed in cases where systemic antibiotic therapy is failing [16]. This was the commonest repeated microbiology investigation with two centres seemingly performing this weekly for each of their five patients over 12 weeks. 

NICE [17] recommends systemic oral antibiotics to be considered if IV antibiotics are not required, based on the severity of infection. This case review identified variation in the mode of systemic antibiotic delivery; 14 (35%) patients had oral and 26 (65%) patients had IV therapy (Table 2). A limitation of this case review is that MDFT centres were not asked to report their reasoning for selected mode of systemic antibiotics and IV therapy may have been considered due to clinical severity. Furthermore, if organisms identified that would only respond to IV antibiotics was not established. Determining whether patient choice was also a factor in decision making was also not explored. Variation was also identified amongst centres on their mode of delivery when treating DFO in same anatomical bones of the foot. DFO located at the same anatomical sites was treated differently with single or dual antibiotic delivery. Therefore, there is a requirement to standardise treatment of specific anatomical sites of DFO and when single or dual systemic therapy may be considered as this has not been addressed in any previous guidelines. 

The duration of systemic antibiotic therapy showed considerable inter-centre variability. NICE [17] and British National Formulary (BNF) [31] recommend a course length is based on clinical assessment and up to 6 weeks for DFO. The NICE [17] guidelines also recommend, based on clinical severity after 48 h of IV therapy, that clinicians consider oral antibiotics for prolonged treatment. The IWGDF [8] recommends treating DFO with systemic antibiotics for up to 6 weeks. 

Systemic antibiotic therapy is known to have toxic effects on renal function with significant reduction in estimated glomerular filtration rate (eGFR) [32]. Four (10%) patients from this case review were identified to develop acute kidney injury (AKI) (Table 3 and Table 12). Liver impairment and injury can also be caused by antibiotic therapy [33]. The most common complication of systemic antibiotic therapy was an abnormal liver test in six (15%) patients (Table 3). Two (5%) of these patients were treated with combined oral therapy clindamycin and ciprofloxacin and two (5%) patients had IV teicoplanin alone. Two (5%) abnormal liver tests and two (5%) AKI occurred in patients on IV teicoplanin therapy (Table 3). Further guidance on systemic antibiotic therapy for patients with existing hepatic or renal impairment is required when treating DFO. A known risk of systemic antibiotic therapy is clostridium difficile (C.diff), and this case review identified no reported cases (Table 12). 

At the end of the 12-week period, 12 (30%) patients were continuing with systemic antibiotic therapy (Table 11). The total number of patients who had clinical quiescence of DFO was 27 (67.5%), and from this group, 5 (12.5%) remained on systemic antibiotic therapy at 12 weeks (Table 11). This was not in accordance with NICE [17] guidelines. Seventeen (42.5%) patients had complete clinical quiescence of DFO with systemic antibiotics alone without surgical intervention, of which nine (22.5%) patients had no recurrence of DFO (Table 11). Prolonged antibiotic therapy has the potential to induce antimicrobial resistance [34]. The practice of prolonging antibiotics as precaution in the absence of infection shown clinically, radiologically or by laboratory investigations must discontinue.

The selection of systemic antibiotic agent, in single or combined form, dosages and mode of delivery was varied amongst all centres. Only one centre (MDFT 2) treated all patients with oral antibiotics (Table 2). It is acceptable an antibiotic choice may have been based on patient factors, drug interactions, previous infections and complications, resistant pathogens and MCS. However, not all centres reported that their antibiotic selection and mode of delivery were aligned with those recommended by NICE [17]. Five centres based systemic antibiotic therapy to treat DFO on their NHS trusts service protocol. Three centres reported having no service protocol. The variation amongst centres in antibiotic choice and mode of delivery may have been due to service limitations, access or dependence on local microbiology data including resistance patterns. An example is that not all services may have had access to IV clinics or hospital at home services. Research focussing on clinical presentation, anatomical location of DFO, patient factors, selective systemic antibiotic regimes in single or combined form, dosages, mode of delivery and duration and outcomes may support future guidelines to develop. 

NICE [17] does not state any haematological, biochemical or laboratory test or diagnostic figure to measure effectiveness when treating DFO with systemic antibiotics. IWGDF [8] guidelines identify diagnostic laboratory tests that can be used to support clinical examinations when assessing the presence of DFO. These tests include blood tests and inflammatory markers such as white blood cells (WBCs), erythrocyte sedimentation rate (ESR), c-reactive protein (CRP) and procalcitonin (PCT). Inflammatory markers CRP and ESR are known diagnostics when treating DFO and to distinguish difference between soft tissue and bone infection [35]. A recent systematic review reported the most accurate inflammatory markers for DFO are CRP and PCT [36].

Based on the significant variations identified in haematological, biochemical and laboratory investigations of this clinical review, there is a requirement for the NICE [17] guidelines to be updated and report when they should be conducted. These essential diagnostics must be appropriately requested based on clinical reasoning and justification when treating DFO with systemic antibiotics.

NICE [17] does not specify how often imaging investigations should be performed. X-ray is recommended to determine the extent of the complication and when DFO is suspected. When X-ray does not show DFO and there is high clinical suspicion, an MRI should be considered [17]. Interval X-rays should be considered for comparison. Of the total 68 number of radiological tests performed by each MDFT centre, 57 (83.8%) were X-rays (Table 5). Thirty-seven (92.5%) patients had an X-ray and ten (25%) underwent MRI investigation (Table 4). All patients with suspected DFO should have an X-ray. Although there is no recommendation on the mode or number of imaging that should be conducted when systemic antibiotics is initiated, a minimum standard and frequency of number of X-rays will prevent unnecessary requests and over exposure of radiation.

The limitations of this case review include the number of centres and number of patients included. The systemic antibiotic interventions for treating DFO in this cohort of patients may not be a true representation of all patients in England and Wales. Another limitation is the lack of additional data that could have provided further insight, such as understanding the access to services and other factors such as specific members and experiences of the MDFT, advice and quality, and standards of wound care. Further patient-related factors may have helped understand why certain patients failed therapy for example poor HbA1c control, smoking or additional co-morbidities. Non-compliance, delayed or missed dosages of antibiotics, or patients failing to attend review appointments were also not recorded. However, this case review has demonstrated there is variation in the current management of DFO with systemic antibiotic therapy, which will support the need for guidelines to be developed. Future research will benefit from exploring the clinical decision making of healthcare providers and the experience of service users on challenges and best practice in the management of DFO.

## 6. Conclusions

The current management of DFO with systemic antibiotics shows there is existing variation in systemic antibiotic therapy regime selection, duration and mode of delivery, measures for effectiveness and outcomes. National guidance is required to inform MDFT teams of standardised care and evidence-based management of DFO.

## Figures and Tables

**Table 1:** Characteristics of patients and location(s) of DFO.

Characteristic	Total Population *n* (%)	MDFT Centre 1	MDFT Centre 2	MDFT Centre 3	MDFT Centre 4	MDFT Centre 5	MDFT Centre 6	MDFT Centre 7	MDFT Centre 8
**Patients** **(*n* = 40)**	40	5	5	5	5	5	5	5	5
**Male** ***n* (%)**	34 (85%)	5 (100%)	5 (100%)	4 (80%)	5 (100%)	4 (80%)	3 (60%)	4 (80%)	4 (80%)
**Female** ***n* (%)**	6 (15%)	0 (0%)	0 (0%)	1 (20%)	0 (0%)	1 (20%)	2 (40%)	1 (20%)	1 (20%)
**Age at diagnosis of DFO, years, mean (SD)**	Mean 62.3 (SD 13.0)	59.4 (SD 9.2)	71.6 (SD 8.2)	53.4 (SD 14.6)	68.2 (SD 13.2)	62.6 (SD 14.7)	64.6 (SD 12.4)	60.6 (SD 9.6)	58.2 (SD 10.7)
**Diabetes type I, II or other**	Type 1 8 (20%) Male = 6 (15%) Female = 2 (5%) Type 2 31 (77.5%) Male = 27 (67.5%) Female = 4 (10%) Other 1 (2.5%) Male = 1 (1%) Female = 0 (0%)	Type 1 1 (20%) Type 2 4 (80%)	Type 1 0 (0%) Type 2 5 (100%)	Type 1 2 (40%) Type 2 3 (60%)	Type 1 0 (0%) Type 2 5 (100%)	Type 1 1 (20%) Type 2 4 (80%)	Type 1 1 (20%) Type 2 4 (80%)	Type 1 0 (0%) Type 2 4 (%) Other 1 (20%)	Type 1 3 (60%) Type 2 2 (40%)
**DFO: Digit**	33 (80.5%)	4	3	5	4	4	5	4	4
**DFO: Metatarsals**	5 (12.2%)	1	3	0	0	0	0	0	1
**DFO: Mid-foot**	2 (4.9%)	0	0	0	1	0	0	1	0
**DFO: Calcaneus**	1 (2.4%)	0	0	0	0	1	0	0	0

**Table 2:** Through which route were systemic antibiotics given for the first time during the current episode of DFO?

	TOTAL	MDFT Centre 1	MDFT Centre 2	MDFT Centre 3	MDFT Centre 4	MDFT Centre 5	MDFT Centre 6	MDFT Centre 7	MDFT Centre 8
**Oral**	26 (65%)	3 (60%)	5 (100%)	4 (80%)	3 (60%)	2 (40%)	3 (60%)	4 (80%)	2 (40%)
**IV**	14 (35%)	2 (40%)	0 (0%)	1 (20%)	2 (40%)	3 (60%)	2 (40%)	1 (20%)	3 (60%)

**Table 3:** Antibiotic(s) frequency prescribed to treat DFO and adverse reactions.

Antibiotic(s) and Mode of Delivery	Frequency (*n* = 40)	Mild Allergic Reaction (Sensitivity) to Antibiotic(s)	Severe Allergic Reaction (Anaphylaxis) to Antibiotic(s)	Acute Kidney Injury (AKI)	Abnormal Liver Test	Clostridium Difficile
Ceftriaxone IV	1	0	0	0	0	0
Ciprofloxacin oral	2	0	0	0	0	0
Clindamycin oral	1	0	0	0	0	0
Clindamycin and ciprofloxacin oral	9	2 (5%)	0	1 (2.5%)	2 (5%)	0
Clindamycin and meropenem IV	1	0	0	0	0	0
Co-Amoxiclav oral	2	0	0	0	0	0
Co-Amoxiclav IV	3	0	0	0	0	0
Co-Amoxiclav and amoxicillin oral	4	0	0	0	1 (2.5%)	0
Co-trimoxazole IV	1	0	0	0	0	0
Doxycycline oral	1	1 (2.5%)	0	0	0	0
Ertapenem and metronidazole IV	1	1 (2.5%)	0	0	0	0
Ertapenem and clindamycin IV	1	0	0	0	0	0
Flucloxacillin oral	3	1 (2.5%)	0	0	0	0
Flucloxacillin and fusidic acid oral	3	0	0	0	0	0
Flucloxacillin and metronidazole IV	1	0	0	1 (2.5%)	1 (2.5%)	0
Linezolid oral	1	0	0	0	0	0
Piperacillin/tazobactam IV	2	0	0	0	0	0
Teicoplanin IV	3	0	0	2 (5%)	2 (5%)	0
**Total**	40	5 (12.5%)	0	4 (10%)	6 (15%)	0

**Table 4:** Which investigation(s) were conducted by the MDFT to determine if the correct antibiotic(s) were given or further antibiotic(s) were needed for the current episode of DFO?

	TOTAL N (%)	MDFT Centre 1 N (%)	MDFT Centre 2 N (%)	MDFT Centre 3 N (%)	MDFT Centre 4 N (%)	MDFT Centre 5 N (%)	MDFT Centre 6 N (%)	MDFT Centre 7 N (%)	MDFT Centre 8 N (%)
**X-ray**	37 (92.5%)	5 (100%)	4 (80%)	5 (100%)	5 (100%)	3 (60%)	5 (100%)	5 (100%)	5 (100%)
**MRI**	10 (25%)	2 (40%)	3 (60%)	1 (20%)	0 (0%)	1 (20%)	1 (20%)	1 (20%)	1 (20%)
**CT**	1 (2.5%)	1 (20%)	0 (0%)	0 (0%)	0 (0%)	0 (0%)	0 (0%)	0 (0%)	0 (0%)
**SPECT-CT**	0 (0%)	0 (0%)	0 (0%)	0 (0%)	0 (0%)	0 (0%)	0 (0%)	0 (0%)	0 (0%)
**18F-FDG PET/CT**	0 (0%)	0 (0%)	0 (0%)	0 (0%)	0 (0%)	0 (0%)	0 (0%)	0 (0%)	0 (0%)
**Radioisotope bone scan**	0 (0%)	0 (0%)	0 (0%)	0 (0%)	0 (0%)	0 (0%)	0 (0%)	0 (0%)	0 (0%)

**Table 5:** How often were these investigations performed within the first 12 weeks following commencement of systemic antibiotics for the first time during the current episode of DFO?

	Total Number of Tests N (%)	MDFT Centre 1 N	MDFT Centre 2 N	MDFT Centre 3 N	MDFT Centre 4 N	MDFT Centre 5 N	MDFT Centre 6 N	MDFT Centre 7 N	MDFT Centre 8 N
**X-ray**	57 (83.8%)	6	1	8	8	3	1	16	14
**MRI**	10 (14.7%)	2	3	1	0	1	1	1	1
**CT**	1 (1.4%)	1	0	0	0	0	0	0	0
**SPECT-CT**	0 (0%)	0	0	0	0	0	0	0	0
**18F-FDG PET/CT**	0 (0%)	0	0	0	0	0	0	0	0
**Radioisotope bone scan**	0 (0%)	0	0	0	0	0	0	0	0
**Total number of investigations**	**68**	**9**	**4**	**9**	**8**	**4**	**2**	**17**	**15**

**Table 6:** Which investigation(s) were conducted by the MDFT to determine if the correct antibiotic(s) were given or further antibiotic(s) were needed for the current episode of DFO?

	Total N (%)	MDFT Centre 1 N (%)	MDFT Centre 2 N (%)	MDFT Centre 3 N (%)	MDFT Centre 4 N (%)	MDFT Centre 5 N (%)	MDFT Centre 6 N (%)	MDFT Centre 7 N (%)	MDFT Centre 8 N (%)
**FBC**	**32 (80%)**	5 (100%)	0 (0%)	5 (100%)	5 (100%)	4 (80%)	3 (60%)	5 (100%)	5 (100%)
**eGFR**	**34 (85%)**	5 (100%)	0 (0%)	5 (100%)	5 (100%)	5 (100%)	4(80%)	5 (100%)	5 (100%)
**U&E profile**	**28 (70%)**	5 (100%)	0 (0%)	5 (100%)	5 (100%)	4 (80%)	4 (80%)	5 (100%)	5 (100%)
**ESR**	**14 (35%)**	0 (0%)	0 (0%)	1 (20%)	3 (60%)	2 (40%)	3 (60%)	0 (0%)	5 (100%)
**CRP**	**33 (82.5%)**	5 (0%)	0 (0%)	5 (100%	5 (100%)	5 (100%)	3 (60%)	5 (100%)	5 (100%)
**LFT**	**31 (77.5%)**	4 (80%)	0 (0%)	5 (100%)	3 (60%)	5 (100%)	4 (80%)	5 (100%)	5 (100%)
**Bicarbonate**	**10 (25%)**	0 (0%)	0 (0%)	0 (0%)	0 (0%)	0 (0%)	0 (0%)	5 (100%)	5 (100%)
**Procalcitonin**	**0 (%)**	0 (0%)	0 (0%)	0 (0%)	0 (0%)	0 (0%)	0 (0%)	0 (0%)	0 (0%)

**Table 7:** How often were these investigations performed within the first 12 weeks following commencement of systemic antibiotics for the first time during the current episode of DFO?

	Total N (%)	MDFT Centre 1 N	MDFT Centre 2 N	MDFT Centre 3 N	MDFT Centre 4 N	MDFT Centre 5 N	MDFT Centre 6 N	MDFT Centre 7 N	MDFT Centre 8 N
**FBC**	226 (19%)	72	0	12	27	24	12	28	51
**eGFR**	161 (13.4%)	81	0	14	28	24	14	0	0
**U&E profile**	241 (20.1%)	77	0	14	28	24	15	34	49
**ESR**	56 (4.6%)	0	0	3	19	0	2	0	32
**CRP**	234 (19.5%)	72	0	13	28	22	14	36	49
**LFT**	232 (19.3%)	72	0	14	27	24	14	36	45
**Bicarbonate**	49 (4.1%)	0	0	0	0	0	0	36	13
**Procalcitonin**	0 (0%)	0	0	0	0	0	0	0	0
**Total tests**	**1199**	**374**	**0**	**70**	**157**	**118**	**71**	**170**	**239**

**Table 8:** Which investigation(s) were conducted by the MDFT to determine if the correct antibiotic(s) were given or further antibiotic(s) were needed for the current episode of DFO?

	Total N (%)	MDFT Centre 1 N (%)	MDFT Centre 2 N (%)	MDFT Centre 3 N (%)	MDFT Centre 4 N (%)	MDFT Centre 5 N (%)	MDFT Centre 6 N (%)	MDFT Centre 7 N (%)	MDFT Centre 8 N (%)
**Wound swab for microscopy culture and sensitivity**	**29 (72.5%)**	5 (100%)	2 (40%)	5 (100%)	5 (100%)	1 (20%)	1 (20%)	5 (100%)	5 (100%)
**Tissue sample for microscopy culture and sensitivity**	**14 (35%)**	1 (20%)	0 (0%)	0 (0%)	3 (60%)	4 (80%)	2 (40%)	0 (0%)	4 (80%)
**Fluid aspirate for microscopy culture and sensitivity**	**0 (0%)**	0 (0%)	0 (0%)	0 (0%)	0 (0%)	0 (0%)	0 (0%)	0 (0%)	0 (0%)
**Bone sample for microscopy culture and sensitivity**	**8 (20%)**	3 (60%)	0 (0%)	0 (0%)	1 (20%)	2 (40%)	0 (%)	2 (40%)	0 (0%)
**Bone sample for histological examination**	**1 (2.5%)**	0 (0%)	0 (0%)	0 (0%)	0 (%)	0 (0%)	0 (0%)	0 (0%)	1 (20%)
**Thermographic scan of the foot with DFO**	**0 (0%)**	0 (0%)	0 (0%)	0 (0%)	0 (0%)	0 (0%)	0 (0%)	0 (0%)	0 (0%)
**Clinician assessment**	**40 (100%)**	5 (100%)	5 (100%)	5 (100%)	5 (100%)	5 (100%)	5 (100%)	5 (100%)	5 (100%)

**Table 9:** How often were these investigations performed within the first 12 weeks following commencement of systemic antibiotics for the first time during the current episode of DFO?

	Total N (%)	MDFT Centre 1	MDFT Centre 2	MDFT Centre 3	MDFT Centre 4	MDFT Centre 5	MDFT Centre 6	MDFT Centre 7	MDFT Centre 8
**Wound swab for microscopy culture and sensitivity**	88 (17.5%)	31	3	5	11	0	0	6	32
**Tissue sample for microscopy culture and sensitivity**	8 (1.6%)	2	0	0	3	2	0	0	1
**Fluid aspirate for microscopy culture and sensitivity**	0 (0%)	0	0	0	0	0	0	0	0
**Bone sample for microscopy culture and sensitivity**	8 (1.6%)	3	0	0	1	2	0	2	0
**Bone sample for histological examination**	1 (0.2%)	0	0	0	0	0	0	0	1
**Thermographic scan of the foot with DFO**	0 (0%)	0	0	0	0	0	0	0	0
**Clinician assessment**	396 (78.8%)	106	17	40	67	33	20	60	53
**Total**	501	143	20	45	82	36	20	69	87

**Table 10:** What was the outcome at 12 weeks following commencement of systemic antibiotics for the first time during the current episode of DFO?

Outcome	Total N (%)	MDFT Centre 1	MDFT Centre 2	MDFT Centre 3	MDFT Centre 4	MDFT Centre 5	MDFT Centre 6	MDFT Centre 7	MDFT Centre 8
**Systemic antibiotics continued**	**12 (30%)**	1 (20%)	2 (40%)	0 (0%)	1 (20%)	1 (20%)	2 (40%)	3 (60%)	2 (40%)
**Systemic antibiotics stopped**	**28 (70%)**	4 (80%)	3 (60%)	5 (100%)	4 (80%)	4 (80%)	3 (60%)	2 (40%)	3 (60%)
**Quiescence of DFO**	**27 (67.5%)**	5 (100%)	2 (40%)	5 (100%)	4 (80%)	4 (80%)	2 (40%)	2 (40%)	3 (60%)
**No recurrence of DFO**	**21 (52.5%)**	4 (80%)	4 (80%)	4 (80%)	4 (80%)	0 (0%)	1 (20%)	3 (60%)	1 (20%)
**Surgery debridement only**	**6 (15%)**	1 (20%)	0 (0%)	0 (0%)	1 (20%)	4 (80%)	0 (0%)	0 (0%)	0 (0%)
**Surgery minor amputation**	**12 (30%)**	2 (40%)	0 (0%)	1 (20%)	1 (20%)	3 (60%)	0 (0%)	2 (40%)	3 (60%)
**Surgery major amputation**	**0 (0%)**	0 (0%)	0 (0%)	0 (0%)	0 (0%)	0 (0%)	0 (0%)	0 (0%)	0 (0%)
**Surgery with biological agent impregnated with antibiotics**	**3 (7.5%)**	1 (20%)	0 (0%)	0 (0%)	0 (0%)	0 (0%)	0 (0%)	0 (0%)	2 (40%)
**Death due to DFO**	**0 (0%)**	0 (0%)	0 (0%)	0 (%)	0 (0%)	0 (0%)	0 (0%)	0 (0%)	0 (0%)

**Table 11:** Systemic antibiotic intervention and outcome at 12-weeks.

Antibiotic(s) and Mode of Delivery	Patients (*n* = 40)	Systemic Antibiotics Continued	Systemic Antibiotics Stopped	Quiescence of DFO	No Recurrence of DFO	Surgery Debridement Only	Surgery Minor Amputation	Surgery Major Amputation	Surgery with Biological Agent Impregnated with Antibiotics	Death
**Ceftriaxone IV**	1	0	1	1	1	0	0	0	0	0
**Ciprofloxacin oral**	2	0	2	1	1	1	0	0	0	0
**Clindamycin oral**	1	1	0	0	1	0	0	0	0	0
**Clindamycin and ciprofloxacin oral**	9	2	7	6	5	0	3	0	0	0
**Clindamycin and meropenem IV**	1	1	0	1	0	0	1	0	1	0
**Co-Amoxiclav oral**	2	2	1	1	0	0	1	0	0	0
**Co-Amoxiclav IV**	3	0	2	2	0	3	2	0	0	0
**Co-Amoxiclav and amoxicillin oral**	4	3	2	1	1	0	1	0	0	0
**Co-Trimoxazole IV**	1	0	1	0	0	0	0	0	0	0
**Doxycycline oral**	1	0	0	0	0	0	1	0	1	0
**Ertapenem and metronidazole IV**	1	0	1	1	1	0	0	0	0	0
**Ertapenem and clindamycin IV**	1	0	1	1	1	0	0	0	0	0
**Flucloxacillin oral**	3	1	2	2	3	0	1	0	0	0
**Flucloxacillin and fusidic acid oral**	3	0	3	3	3	0	1	0	0	0
**Flucloxacillin and metronidazole IV**	1	0	1	1	1	1	0	0	0	0
**Linezolid oral**	1	0	1	1	1	0	0	0	0	0
**Piperacillin/tazobactam IV**	2	1	1	2	1	0	0	0	0	0
**Teicoplanin IV**	3	1	2	3	1	1	1	0	1	0
**TOTAL**	**40**	**12 (30%)**	**28 (70%)**	**27 (67.5%)**	**21 (52.5%)**	**6 (15%)**	**12 (30%)**	**0 (%)**	**3 (7.5%)**	**0 (0%)**

**Table 12:** Did any of the following complications occur within 12 weeks following commencement of systemic antibiotics for the first time during the current episode of DFO?

	Total N (%)	MDFT Centre 1 N (%)	MDFT Centre 2 N (%)	MDFT Centre 3 N (%)	MDFT Centre 4 N (%)	MDFT Centre 5 N (%)	MDFT Centre 6 N (%)	MDFT Centre 7 N (%)	MDFT Centre 8 N (%)
**Mild allergic reaction (sensitivity) to antibiotic(s)**	**5 (12.5%)**	0 (%)	1 (20%)	1 (20%)	0 (0%)	0 (0%)	0 (0%)	2 (40%)	1 (20%)
**Severe allergic reaction (anaphylaxis) to antibiotic(s)**	**0 (0%)**	0 (0%)	0 (0%)	0(%)	0 (0%)	0 (0%)	0 (0%)	0 (0%)	0 (%)
**Acute kidney injury (AKI)**	**4 (10%)**	1 (20%)	0 (0%)	1 (20%)	1 (20%)	0 (0%)	0 (0%)	0 (0%)	1 (20%)
**Abnormal liver test**	**6 (15%)**	2 (40%)	0 (0%)	2 (40%)	1 (20%)	0 (0%)	1 (20%)	0 (0%	0 (0%)
**Clostridium difficile**	**0 (0%)**	0 (0%)	0 (0%)	0 (0%)	0 (0%)	0 (0%)	0 (0%)	0 (0%)	0 (0%)
**Total**	**15**	3	1	4	2	0	1	2	1

## Data Availability

The original contributions presented in the study are included in the article, further enquiries can be directed to the corresponding author.
